# Evaluating the combined toxicity of *Penicillium* mycotoxins in absorption, metabolism, and excretion systems

**DOI:** 10.1002/jsfa.70686

**Published:** 2026-05-11

**Authors:** Carolina Sousa Monteiro, Eugénia Pinto, Miguel A Faria, Sara C Cunha

**Affiliations:** ^1^ LAQV‐REQUIMTE, Laboratory of Bromatology and Hydrology, Faculty of Pharmacy University of Porto Porto Portugal; ^2^ Laboratory of Microbiology, Biological Sciences Department Faculty of Pharmacy of University of Porto Porto Portugal; ^3^ Interdisciplinary Centre of Marine and Environmental Research (CIIMAR/CIMAR) University of Porto Matosinhos Portugal

**Keywords:** mycotoxins cytotoxicity, toxicological interactions, NCI‐N87 cells, HepG2 cells, Caco‐2 cells, HEK‐293 cells

## Abstract

**BACKGROUND:**

*Penicillium* mycotoxins, including ochratoxin A (OTA), citrinin (CIT), and cyclopiazonic acid (CPZ), frequently co‐occur in food commodities, contributing to chronic low‐level dietary exposure. However, current risk assessments often consider these toxins individually, overlooking potential interaction effects. This study evaluated the individual and combined cytotoxicity of OTA, CIT, and CPZ using human cell models representing key barriers of absorption (gastric and intestinal), metabolism (hepatic) and excretion (renal). The combination index–isobologram method was applied to characterize interaction dynamics.

**RESULTS:**

Ochratoxin A exhibited the highest toxicity across all cell types, followed by CPZ and CIT. Binary mixtures revealed strong synergistic interactions, particularly OTA:CPZ in liver cells and OTA:CIT in liver and kidney cells. Ternary mixtures primarily affected kidneys. High dose reduction index (DRI) values were observed at low inhibition levels, reaching 21.82‐fold, indicating enhanced toxicity potential at dietary‐relevant concentrations. Synergism was most pronounced at low inhibition levels (IC_10_), representative of chronic dietary exposure, emphasizing the risk of real‐world co‐exposure.

**CONCLUSION:**

These results demonstrate that low‐dose synergic interactions among *Penicillium* mycotoxins may enhance toxic risk under realistic exposure scenarios. Incorporating mixture effects into food safety risk assessment frameworks is therefore essential to better reflect chronic human exposure conditions. © 2026 The Author(s). *Journal of the Science of Food and Agriculture* published by John Wiley & Sons Ltd on behalf of Society of Chemical Industry.

**ABBREVIATIONS**
CI: combination index
CIT: citrinin
CM: culture medium
CPZ: cyclopiazonic acid
DMEM: Dulbecco's modified Eagle's medium
DRI: dose reduction index
Fa: fraction affected
FBS: fetal bovine serum
IC: inhibitory concentration
MEM NEAA: minimum essential medium non‐essential amino acids
MTT: 3‐(4,5‐dimethylthiazol‐2‐yl)‐2,5‐diphenyltetrazolium bromide
OTA: ochratoxin A
RPMI: Roswell Park Memorial Institute medium
TDI: tolerable daily intake

## INTRODUCTION

Humans can be exposed to mycotoxins through multiple pathways, including inhalation of contaminated air, ingestion of contaminated food and water, and dermal contact.^1^, ^2^ Environmental contamination plays a critical role in the introduction of mycotoxins into the food chain. Climate change, shifting agricultural practices, and post‐harvest storage conditions further promote fungal growth and toxin biosynthesis, increasing the likelihood of multi‐mycotoxin exposure.^3^, ^4^ These compounds are chemically stable and resistant to many processing conditions, contributing to their persistence throughout the food chain.^5^, ^6^

*Penicillium* mycotoxins are recurrent contaminants of stored foods and feeds and represent a persistent global food safety challenge. Among them, ochratoxin A (OTA), citrinin (CIT), and cyclopiazonic acid (CPZ) are frequently detected in cereals and cereal‐based products, and often co‐occur.^7^, ^8^, ^9^, ^10^ Despite this documented co‐occurrence in food, current risk‐assessment frameworks largely evaluate these toxins individually, overlooking potential interaction effects that may arise under realistic co‐exposure scenarios. This limitation is particularly concerning given that human exposure is typically chronic and at low levels, contributing to the overall exposome.

Ochratoxin A is a regulated mycotoxin with established limits due to its known health risks. It causes nephrotoxicity primarily by interfering with cellular metabolism, inhibiting protein synthesis, inducing oxidative stress, and damaging mitochondria. These effects may lead to DNA damage, apoptosis, and, with prolonged exposure, potentially carcinogenic outcomes. The International Agency for Research on Cancer (IARC) classifies OTA as a Group 2B carcinogen (possibly carcinogenic to humans).^2^, ^11^, ^12^, ^13^ In contrast, CIT and CPZ are considered emerging or insufficiently characterized contaminants. They lack regulatory limits but are causing growing concern.^7^, ^8^, ^9^, ^14^, ^15^

Citrinin, like OTA, targets renal cells by impairing mitochondrial respiration and promoting oxidative damage, and has been linked to Balkan endemic nephropathy.^10^, ^16^ Although detected in food, its health impact remains unclear, and IARC places it in Group 3 (not classifiable as to its carcinogenicity to humans).^11^, ^12^, ^17^, ^18^

Cyclopiazonic acid, which has been studied less, has been described as a potent inhibitor of sarco/endoplasmic reticulum Ca^2+^‐ATPase (SERCA). It disrupts calcium homeostasis, triggering cellular stress responses that affect hepatic, renal, and gastrointestinal tissues in animals.^19^, ^20^ Its acute toxicity is low but chronic exposure may cause neurotoxicity and other degenerative effects, underscoring the need for further toxicological evaluation.^21^, ^22^, ^23^ Due to the lack of toxicity data, the IARC Monographs have not yet considered this toxin.^12^, ^23^ Continued research is crucial to understand CPZ occurrence, interactions with co‐occurring mycotoxins, and their health effects on humans and animals. Although their individual toxicities are recognized, their combined effects remain poorly characterized. These partially overlapping but distinct molecular mechanisms raise the possibility that combined exposure could amplify cellular stress pathways, overwhelm detoxification systems, or alternatively compete for transporters and metabolic enzymes, resulting in synergistic or antagonistic interactions.^10^, ^24^, ^25^, ^26^, ^27^, ^28^

From a regulatory perspective, the European Food Safety Authority (EFSA) has emphasized the importance of harmonized methodologies for assessing combined exposure to multiple chemicals.^10^, ^15^, ^24^, ^29^ However, experimental data supporting mixture risk assessment for *Penicillium* mycotoxins remain scarce. Most toxicological studies focus on high‐dose, single‐compound effects, despite evidence that chronic dietary exposure occurs at lower concentrations where interaction dynamics may differ substantially.^8^, ^24^ Importantly, low‐level synergistic interactions could reduce effect thresholds and challenge current tolerable daily intake (TDI) frameworks that assume dose additivity.

The aim of this study was to elucidate the organ‐specific and interaction‐driven cytotoxic effects of three co‐occurring *Penicillium* mycotoxins – OTA, CIT, and CPZ – particularly under conditions relevant to chronic dietary exposure. Using human cell models representing absorption (gastric and intestinal), metabolism (hepatic), and excretion (renal), the individual and combined interactions were characterized quantitatively through the combination index–isobologram approach. This facilitated the determination of interaction types (synergistic, additive, or antagonistic) and established whether co‐exposure would modify toxicity magnitude and target organ susceptibility, representing a novel contribution to this field. By integrating mixture toxicology with organ‐specific cellular models, this study addresses a critical gap in food toxicology and provides insight into the health implications of chronic low‐level co‐exposure to *Penicillium* mycotoxins.

## MATERIALS AND METHODS

### Reagents and materials

Cyclopiazonic acid (10 mg, ≥98% purity) and CIT (5 mg, ≥98% purity) were purchased from Santa Cruz Biotechnology (Heidelberg, Germany). Ochratoxin A (1 mg, ≥98% purity) and 0.4% trypan blue stain solution were purchased from Sigma‐Aldrich (St Louis, MO, USA). 3‐(4,5‐Dimethylthiazol‐2‐yl)‐2,5‐diphenyltetrazolium bromide (MTT) and dimethyl sulfoxide (DMSO) were purchased from Duchefa Biochemie (Haarlem, The Netherlands). Roswell Park Memorial Institute medium (RPMI), fetal bovine serum (FBS), 0.25% trypsin solution, minimum essential medium non‐essential amino acids (MEM NEAA) 100×, GlutaMAX 100×, and penicillin/streptomycin (100×; 10 000 units mL⁻¹ and 10 mg mL⁻¹, respectively) were purchased from Gibco (Life Technologies Corporation) (Paisley, United Kingdom). High‐glucose Dulbecco's modified Eagle's medium (DMEM) was purchased from Lonza (Walkersville, MD, USA).

### Mycotoxins solutions

All stock solutions were prepared in methanol and stored at −20 °C. For NCI‐N87 cells assays, stock solutions of citrinin (CIT; 2.032 mg mL⁻¹, 8120 μM), cyclopiazonic acid (CPZ; 1.958 mg mL⁻¹, 5822 μM), and ochratoxin A (OTA; 0.457 mg mL⁻¹, 1131 μM) were used. For HepG2 assays, stock solutions of CIT (1.857 mg mL⁻¹, 7422 μM), CPZ (1.958 mg mL⁻¹, 5822 μM), and OTA (0.457 mg mL⁻¹, 1131 μM) were used. For Caco‐2 and HEK‐293 assays, stock solutions of CIT (3.313 mg mL⁻¹, 13 238 μM), CPZ (1.735 mg mL⁻¹, 5158 μM), and OTA (0.457 mg mL⁻¹, 1131 μM) were used. Before each experiment, working solutions were prepared in a complete cell culture medium (CM).

### Cell culture

Human‐derived gastric cells (NCI‐N87) were provided by the Institute for Research and Innovation in Health of the University of Porto (I3S), Porto, Portugal, and human intestinal cells (Caco‐2) were supplied by the Fisico‐Química Molecular group from the University of Coimbra, Portugal. Hepatic (HepG2) and kidney (HEK‐293) cells were purchased from Leibniz Institute DSMZ‐German Collection of Microorganisms and Cell Cultures, Braunschweig, Germany.

All cells were grown in 75 cm^2^ culture flasks at 37 °C and 5% CO_2_ in cell‐specific CM. NCI‐N87 cells were maintained in RPMI supplemented with 10% heat‐inactivated FBS and 1% penicillin/streptomycin; HepG2 cells were maintained in DMEM supplemented with 10% heat‐inactivated FBS and 1% penicillin/streptomycin. Caco‐2 cells were maintained in DMEM supplemented with 10% heat‐inactivated, 1% penicillin/streptomycin, 1% MEM NEAA, and 1% GlutaMAX. HEK‐293 cells were maintained in DMEM with 10% heat‐inactivated FBS, 1% penicillin/streptomycin, and 1% MEM NEAA. The cells were trypsinized (1 mL, 5 min incubated at 37 °C with 5% CO_2_) at 80% to 90% of confluency and seeded in 96‐well plates (TPP, Trasadingen, Switzerland). The assays were performed between passages 18–26 for NCI‐N87 cells, 79–92 for Caco‐2 cells, 5–20 for HepG2 cells, and 5–16 for HEK‐293 cells.

### Individual and combined effects

The individual toxic effects of each mycotoxin on cell viability were evaluated by exposing cells to a range of concentrations, using twofold serial dilutions, except for CIT on HEK‐293 cells, where a 1.2‐fold dilution series was used. The resulting data were used to fit dose–response curves for each compound and to calculate the IC_50_ values for each cell line.

The combined toxicity was studied in binary (CIT:CPZ, CIT:OTA, and CPZ:OTA) and ternary combinations. Cells were exposed to varying concentrations according to a constant‐ratio design based on the Chou–Talalay method,^30^ implemented at an equipotency ratio (i.e., (IC_50_)_1_/(IC_50_)_2_ ratio) to ensure approximately equal contributions of each compound to the combined effect (Table 1).

**Table 1:** Range of concentrations (μM) used in the assays for each cell line and individual/combined mycotoxins

	NCI‐N87		Caco‐2		HepG2		HEK‐293	
Individual	DF	Dose range (μM)	DF	Dose range (μM)	DF	Dose range (μM)	DF	Dose range (μM)
CIT	2	28.01–80.00	2	20.00–160.00	2	10.00–160.00	1.2	24.11–60.00
CPZ	2	0.63–40.00	2	0.31–20.00	2	0.63–40.00	2	0.94–15.00
OTA	2	0.31–20.00	2	0.39–12.50	2	0.78–25.00	2	0.39–25.00
Combined^a^								
CIT:CPZ	2	5.36–171.60	2	15.14–241.82	2	5.26–84.08	2	3.36–107.60
CIT:OTA	2	4.93–157.60	2	7.35–235.26	2	5.21–83.30	2	2.72–87.14
CPZ:OTA	2	1.69–54.00	2	0.63–20.26	2	0.97–15.54	2	0.82–26.38
CIT:CPZ:OTA	2	5.99–95.80	2	7.73–248.66	2	5.72–182.92	2	3.46–55.28

^a^ The combination concentration range reflects the sum of the individual compound concentrations.

Abbreviations: DF, dilution factor; CIT, citrinin; CPZ, cyclopiazonic acid; OTA, ochratoxin.

### Cell viability assay

The MTT assay was used to evaluate the individual and combined effects of CIT, CPZ, and OTA. NCI‐N87 (2500 cells per well), Caco‐2 (2500 cells per well), HepG2 (2000 cells per well), and HEK‐293 (2000 cells per well) cells were seeded in 96‐well plates and allowed to adhere for 24 h at 37 °C and 5% CO₂. Cells were then exposed to mycotoxins for 72 h, either individually or in binary and ternary combinations. Control groups were treated with CM alone or with CM containing the highest methanol concentration used in the mycotoxin treatments (3.3% for NCI‐N87, 2.6% for Caco‐2, 3.6% for HepG2, and 2.2% for HEK‐293) to confirm cell viability under experimental conditions. After 72 h of exposure, the culture medium was removed, and 100 μL of freshly prepared MTT solution (0.5 mg mL⁻¹) was added to each well. Dimethyl sulfoxide (100 μL) was then added, and absorbance was measured at 570 nm after 30 min using a microplate reader (SPECTROstar Nano, BMG Labtech, Offenburg, Germany).

### Data analyses

All measurements were conducted in six replicates across three independent experiments. Data are presented as means ± standard errors of the mean (SEMs) and were plotted as dose–response curves using GraphPad Prism version 8.0.2 for Windows (GraphPad Software, La Jolla, CA, USA).

Combination ratios were calculated based on equipotent inhibitory concentrations at 50% (IC₅₀) derived from individual exposure experiments, ensuring equal contributions of each toxin to the overall effect. The IC₅₀ values for combination index (CI) analysis were determined using CompuSyn software, version 1.0 (ComboSyn Inc., Paramus, NJ, USA), based on the median‐effect equation of the mass‐action law described by Chou and Talalay.^31^ A CI value between 0.9 and 1.1 indicates an additive effect, whereas CI < 0.9 and CI > 1.1 indicate synergistic and antagonistic effects, respectively.

The dose reduction indices (DRIs) were also calculated and indicate the fold reduction in the dose of each compound in combination required to achieve the same effect as each compound alone.^30^

## RESULTS AND DISCUSSION

### Cytotoxicity of individual mycotoxins

**Figure 1:**
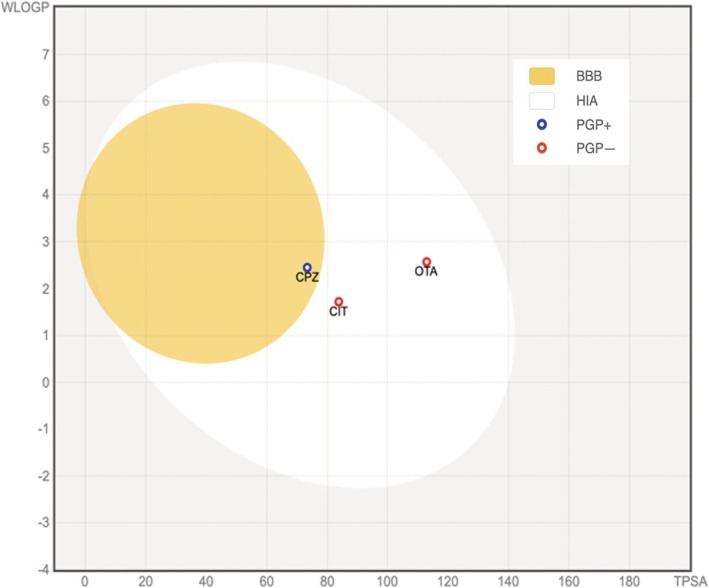
BOILED‐Egg graph for mycotoxins. BBB, blood–brain barrier; HIA, human intestinal absorption; PGP, P‐glycoprotein (blue: substrate for P‐glycoprotein, red: not a substrate for P‐glycoprotein). Source: SwissADME.^32^

The toxicity of three *Penicillium* mycotoxins – OTA, CIT, and CPZ – was assessed individually and in combination across various human organs relevant to absorption, metabolism, and excretion. This includes processes such as nutrient digestion, energy production and use, water balance regulation, and metabolic waste elimination. The NCI‐N87 cell line was chosen as a gastric model, representing the initial barrier encountered by mycotoxins during digestion. The Caco‐2 cell line modeled the intestinal barrier, which is relevant during and after digestion. Based on the BOILED‐Egg method (Fig. 1), all the mycotoxins could cross the intestinal barrier; thus, HepG2 cells were used as a hepatic model, the main metabolic organ and toxicity target for these xenobiotics. Finally, the HEK‐293 cell line was selected as a kidney cellular model due to its crucial role in xenobiotic excretion and the known kidney toxicity of some mycotoxins, namely OTA and CIT. In this model, compounds located in the white region (egg white) indicate a high likelihood of intestinal absorption (e.g., OTA and CIT), while those in the yellow region (egg yolk) suggest potential to cross the blood–brain barrier (e.g., CPZ). Blue circles denote P‐glycoprotein substrates, and red indicate non‐substrates. Compounds in the grey region have a low probability of human intestinal absorption and blood–brain barrier permeation.

**Figure 2:**
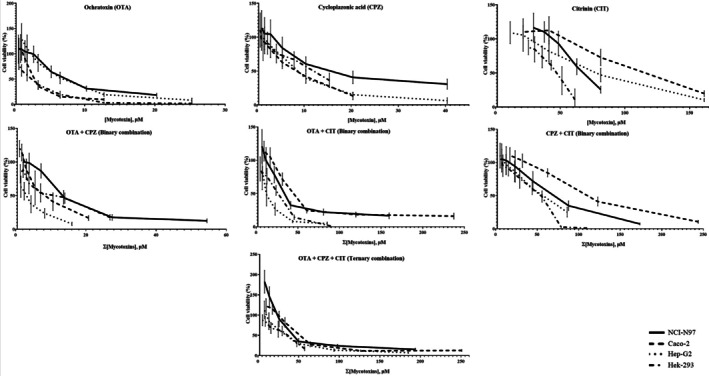
Percentage of cell viability after 72 h exposure to citrinin (CIT), cyclopiazonic acid (CPZ) and ochratoxin A (OTA) individually and in binary and ternary combination with the different cell lines. Concentration data are reported as means ± SEMs of at least three independent experiments.

All mycotoxins (OTA, CIT, and CPZ), when tested individually, produced different dose–response profiles across all cell lines, with corresponding IC_50_ values (Fig. 2). Table 2 presents the parameters (*r, m*, and *D*_*m*_) of the individual toxicity curves, along with the concentrations required to reduce cell viability by 10% (IC_10_), 50% (IC_50_), and 90% (IC_90_).

**Table 2:** Parameters calculated from Compusyn software based on the Chou–Talalay method for individual and combined effects

NCI‐N87					
Individual	*r*	*m*	IC_10_	IC_50_ (Dm)	IC_90_
OTA	0.90	1.70	2.79	10.14	36.85
CPZ	0.94	1.60	4.35	17.09	67.19
CIT	0.97	5.93	47.52	68.85	99.75

Combination	Ratio	*r*	*m*	IC_10_	CI value^a^	IC_50_ (Dm)	CI value^a^	IC_90_	CI value^a^
OTA:CPZ	1:1.7	0.98	2.16	5.99	1.66 A	16.60	1.22 MA	46.01	0.89 sS
OTA:CIT	1:6.9	0.93	2.18	18.12	1.16 sA	49.75	1.25 MA	136.59	1.67 A
CPZ:CIT	1:4	0.98	2.19	23.28	1.46 MA	63.44	1.48 A	172.87	1.90 A
OTA:CPZ:CIT	1:1.7:6.9	0.96	2.13	22.43	2.09 A	62.89	1.95 MA	176.29	2.23 A

Caco‐2					
Individual	*r*	*m*	IC_10_	IC_50_ (Dm)	IC_90_
OTA	0.97	2.27	1.30	3.42	8.99
CPZ	0.99	1.46	1.50	6.71	30.11
CIT	0.95	3.12	56.46	114.21	231.05

Combination	Ratio	*r*	*m*	IC_10_	CI value^a^	IC_50_ (Dm)	CI value^a^	IC_90_	CI value^a^
OTA:CPZ	1:2	0.98	1.96	2.68	1.88 A	8.24	1.62 A	25.31	1.50 A
OTA:CIT	1:33.4	0.93	2.13	23.38	0.92 NA	65.72	1.12 sA	184.73	1.37 MA
CPZ:CIT	1:17.0	0.97	2.66	49.74	2.68 A	113.66	1.88 A	259.75	1.54 A
OTA:CPZ:CIT	1:2:33.4	0.95	2.26	25.51	1.89 A	67.47	1.64 A	178.48	1.58 A

Hep‐G2					
Individual	*r*	*m*	IC_10_	IC_50_ (Dm)	IC_90_
OTA	0.98	2.25	2.78	7.38	19.58
CPZ	0.99	1.77	2.35	8.16	28.28
CIT	0.97	2.63	32.90	75.93	175.19

Combination	Ratio	*r*	*m*	IC_10_	CI value^a^	IC_50_ (Dm)	CI value^a^	IC_90_	CI value^a^
OTA:CPZ	1:1.1	0.98	1.07	0.27	0.11 SS	2.08	0.27 SS	16.15	0.69 S
OTA:CIT	1:10.3	0.98	1.73	3.38	0.20 SS	12.05	0.29 SS	43.00	0.42 S
CPZ:CIT	1:9.3	0.99	2.07	17.67	1.21 MA	51.19	1.22 MA	148.32	1.27 MA
OTA:CPZ:CIT	1:1.1:10.3	0.97	1.97	12.22	1.13 sA	37.27	1.22 MA	113.70	1.37 MA

Hek‐293					
Individual	*r*	*m*	IC_10_	IC_50_ (Dm)	IC_90_
OTA	0.97	1.37	0.30	1.48	7.34
CPZ	0.95	1.01	1.32	11.71	103.21
CIT	0.97	4.26	25.14	42.09	70.48

Combination	Ratio	*r*	*m*	IC_10_	CI value^a^	IC_50_ (Dm)	CI value^a^	IC_90_	CI value^a^
OTA:CPZ	1:7.9	0.94	0.93	0.73	0.76 MS	7.80	1.18 sA	83.61	2.00 A
OTA:CIT	1:28.4	0.93	1.73	3.74	0.57 S	13.27	0.61 S	47.08	0.86 sS
CPZ:CIT	1:3.6	0.95	2.48	11.20	2.18 A	27.13	1.01 NA	65.71	0.87 sS
OTA:CPZ:CIT	1:7.9:28.4	0.94	1.20	3.80	1.06 NA	23.70	1.29 MA	147.92	2.44 A

*r* values represent the goodness‐of‐fit curve; *m* signifies the shape of the dose‐effect curve (*m* = 1, hyperbolic, *m* > 1 sigmoidal, *m* < 1 flat sigmoidal); all inhibition concentrations (IC_10_/ IC_50_/IC_90_) are expressed as μM. The ICs reported in combinations are the sum of the individual ICs when in combination; CI‐combination index.

^a^ Type of interaction: SS, strong synergism; S, synergism; MS, moderate synergism; sS, slight synergism; NA, nearly additive; sA, slight antagonism; MA, moderate antagonism; A, antagonism; SA, strong antagonism.^33^

Based on IC_50_ values, OTA exhibited the highest toxicity across all cell lines, followed by CPZ and CIT. This trend was generally consistent at IC₁₀ and IC₉₀, with two exceptions. In HepG2 cells at IC₁₀, the toxicity ranking was CPZ > OTA > CIT, and in HEK‐293 cells at IC₉₀, the ranking was OTA > CIT > CPZ. These results indicate that CPZ may exert a greater effect on HepG2 cells at lower concentrations.

Regarding the sensitivity of different cell lines to each mycotoxin (Table 2), OTA exhibited the highest toxicity in the following order: HEK‐293 > Caco‐2 > HepG2 > NCI‐N87; for CPZ: Caco‐2 > HepG2 > HEK‐293 > NCI‐N87. For CIT: HEK‐293 > NCI‐N87 > HepG2 > Caco‐2. These results indicate that kidney‐derived cells were the most sensitive to CIT and OTA, whereas the intestinal cells showed greater susceptibility to CPZ.

At low concentrations, the ranking for CPZ and CIT shifted to HEK‐293 > Caco‐2 > HepG2 > NCI‐N87 and HEK‐293 > HepG2 > NCI‐N87 > Caco‐2, respectively, reinforcing the kidneys as the primary target.

Previous studies on mycotoxin toxicity in Caco‐2 cells have focused primarily on individual exposures. Ochratoxin A was the most extensively studied, whereas data for citrinin (CIT) are lacking. When comparing the results of the present study (IC₅₀ = 3.42 μM) with reported values (IC₅₀ = 2.07 μM), similar cell viability outcomes were observed following 72 h exposure to OTA.^34^ Other studies have reported IC₅₀ values ranging from 7.4 to 145.36 μM for OTA; however, these differences likely reflect variations in exposure duration and cell density.^33^, ^35^, ^36^, ^37^ For CPZ, only one study has reported an IC_50_ of more than 0.124 μM compared with 6.71 μM observed in the present study.^38^

In hepatic cells, the IC_50_ for OTA (7.38 μM) falls within the range reported in the literature for 72 h exposure (1.86 to 36 μM).^39^, ^40^ Other studies have reported IC₅₀ values for OTA ranging from 21.16 to 220 μM, likely reflecting differences in exposure time and cell density.^33^, ^41^, ^42^, ^43^ For CIT, greater toxicity was observed in the present study (IC_50_ of 75.93 μM) compared with reported values (IC_50_ of 150–155 μM) following 72 h exposure.^41^, ^42^ No data were identified in the literature for CPZ‐induced cytotoxicity in HepG2 cells.

For renal cells, no studies were identified that evaluated 72 h exposure; however, OTA and CIT have been examined at 24 h exposure. The present study shows greater toxicity for OTA compared with reported values (IC₅₀ = 1.48 μM vs. 16 μM).^44^ For CIT, the IC₅₀ observed in this study (42.09 μM) falls within the range reported in the literature (0.44 to 189 μM).^44^, ^45^ This suggests that these mycotoxins may exert greater effects with prolonged exposure than short‐term exposure. No data were identified for CPZ in HEK‐293 cells. Variability in reported IC₅₀ values may also reflect differences in cell‐line characteristics, such as metabolic activity and antioxidant capacity, as well as experimental conditions, including endpoints measured, exposure duration, and the presence or absence of serum in the culture medium.

In this context, OTA is the most concerning due to its high potency at low concentrations. Although CIT and CPZ appear less toxic per μM, cumulative and combined exposure from food sources underscores the need for further investigation. The results demonstrate marked differences in OTA, CPZ, and CIT across cell types (intestinal, hepatic, and renal) and highlight a notable gap for the NCI‐N87 gastric model. The gastrointestinal epithelium plays a key role as a barrier preventing the entry of foodborne contaminants from the intestinal lumen. Disruption of this barrier can increase permeability, allowing typically excluded compounds to cross and potentially contribute to intestinal disorders. This knowledge gap also extends to most of the mycotoxin combinations tested in the present study, which is addressed in later sections of this paper.

### Cytotoxicity of binary combinations of mycotoxins

**Figure 3:**
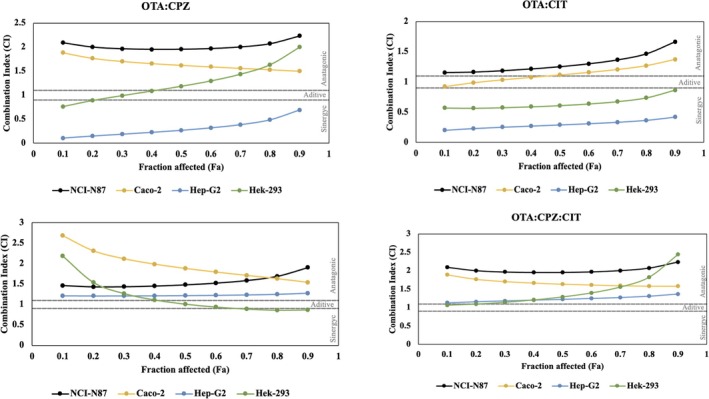
Combination index plots (CI‐Plots) for binary/ternary combinations of mycotoxins based on the combination index theorem after 72 h exposure. The CI values were calculated from data obtained from three independent experiments. CI < 0.90, 0.90 < CI < 1.10, and CI > 1.10 indicate synergistic, additive, and antagonistic effects, respectively^30^. Dashed lines indicate the additive level, which separates synergism and antagonism. CIT, citrinin; CPZ, cyclopiazonic acid; OTA, ochratoxin A.

All binary combinations produced dose‐dependent reductions in cell viability across all cell lines, with greater effects observed at higher concentrations (Fig. 2). The parameters (*r*, *m*, and *D*_m_) of the dose–response curves, together with the concentrations required to reduce cell viability by 10% (IC₁₀), 50% (IC₅₀), and 90% (IC₉₀) for each binary mixture, are presented in Table 2 and Fig. 3.

Most combinations exhibited antagonistic effects across all cell types. However, exceptions were observed for OTA‐containing mixtures, specifically OTA:CPZ in hepatic cells and OTA:CIT in both hepatic and renal cells, where synergism was consistently observed across all concentration levels (Fig. 3). Based on IC₅₀ values, the OTA:CPZ combination was the most toxic across all cell lines, followed by OTA:CIT and CPZ:CIT (Table 2). This pattern was also reflected at IC₁₀ and IC₉₀, except in HEK‐293 cells at IC₉₀, where the toxicity ranking shifted to OTA:CIT > CPZ:CIT > OTA:CPZ (Table 2).

Distinct toxic effects were observed for different toxin combinations. For OTA:CPZ, toxicity followed the order HepG2 > HEK‐293 > Caco‐2 > NCI‐N87; for OTA:CIT, HepG2 > HEK‐293 > NCI‐N87 > Caco‐2; and for CPZ:CIT, HEK‐293 > HepG2 > NCI‐N87 > Caco‐2 (Table 2).

Ochratoxin A alone showed the highest toxicity in renal cells, whereas in combination with CPZ or CIT it exerted greater effects in hepatic cells. Cyclopiazonic acid alone was most toxic in intestinal cells, whereas in combination it showed increased toxicity in renal cells with CIT and in hepatic cells with OTA. CIT exhibited the highest toxicity in renal cells both alone and in combination with CPZ, and in hepatic cells when combined with OTA.

**Figure 4:**
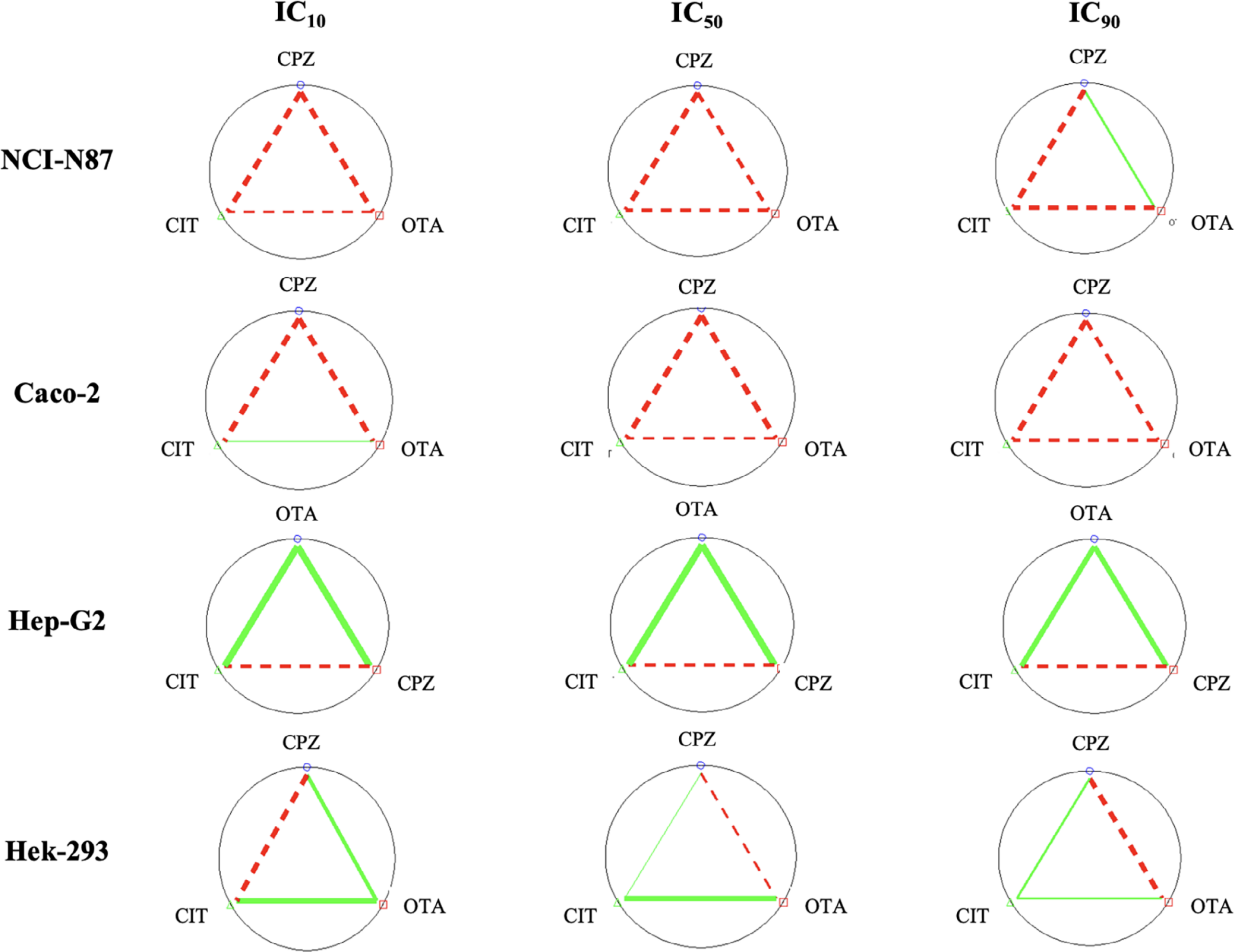
Polygonograms with the type of toxicological interaction of the binary combination. Green solid lines represent  synergistic or additive interactions; while dashed red lines represents antagonistic interactions. All results were calculated using Compusyn software. CIT, citrinin; CPZ, cyclopiazonic acid; OTA, ochratoxin A; IC_10_, inhibition concentration that inhibits 10% of the cell population; IC_50_, inhibition concentration that inhibits 50% of the cell population; IC_90_, inhibition concentration that inhibits 90% of the cell population.

The Chou–Talalay method also enables calculation of CI values, which quantify interaction effects of mycotoxin combinations (synergism: CI < 0.9; additive: 0.9 to 1.1; antagonism: CI > 1.1). Combination index values are presented as CI plots and polygonograms for all cell types and combinations in Figs 3 and 4, respectively.

Although OTA:CPZ was the most toxic combination across all cell lines, synergistic effects were observed consistently only in hepatic cells. In renal cells, the interaction was generally moderate and additive, particularly at low levels of inhibition (Fa < 0.4). Overall, the OTA:CIT combination exhibited the greatest degree of synergism across cell lines, despite not being the most toxic overall. Synergism was observed in both hepatic and renal cells across all inhibition levels, whereas additive and antagonistic effects were observed in intestinal and gastric cells, respectively. The least toxic binary combination, CPZ:CIT, was observed to have an antagonistic effect on gastric, intestinal, and liver cells and a slight synergistic effect in kidney cells at high inhibition levels (Fa > 0.4).

The polygonograms shown in Fig. 4 summarize the distribution of the type and magnitude of interactions amongst the different cell models. More synergic effects (green lines) were detected in HepG2 and HEK‐293 between OTA – a regulated and ubiquitarian mycotoxin – and CPZ or CIT, the emerging ones. The quantity and the magnitude of the synergisms were also higher at IC_10_ concentrations, where the doses are particularly relevant for assessing chronic dietary exposure, in which mycotoxins are present at subtoxic concentrations (Table 2 and Fig. 4). These results highlight the importance of considering the synergistic effects of mycotoxins when evaluating risks associated with food consumption. In gastric and intestinal cells, antagonistic effects were observed consistently across all inhibition levels, indicating reduced toxicity when mycotoxins were combined. This antagonism suggests that when multiple mycotoxins are present, they may compete for cellular uptake, metabolic enzymes, or detoxification pathways, reducing each other's effectiveness in causing cellular damage.^46^, ^47^, ^48^

**Figure 5:**
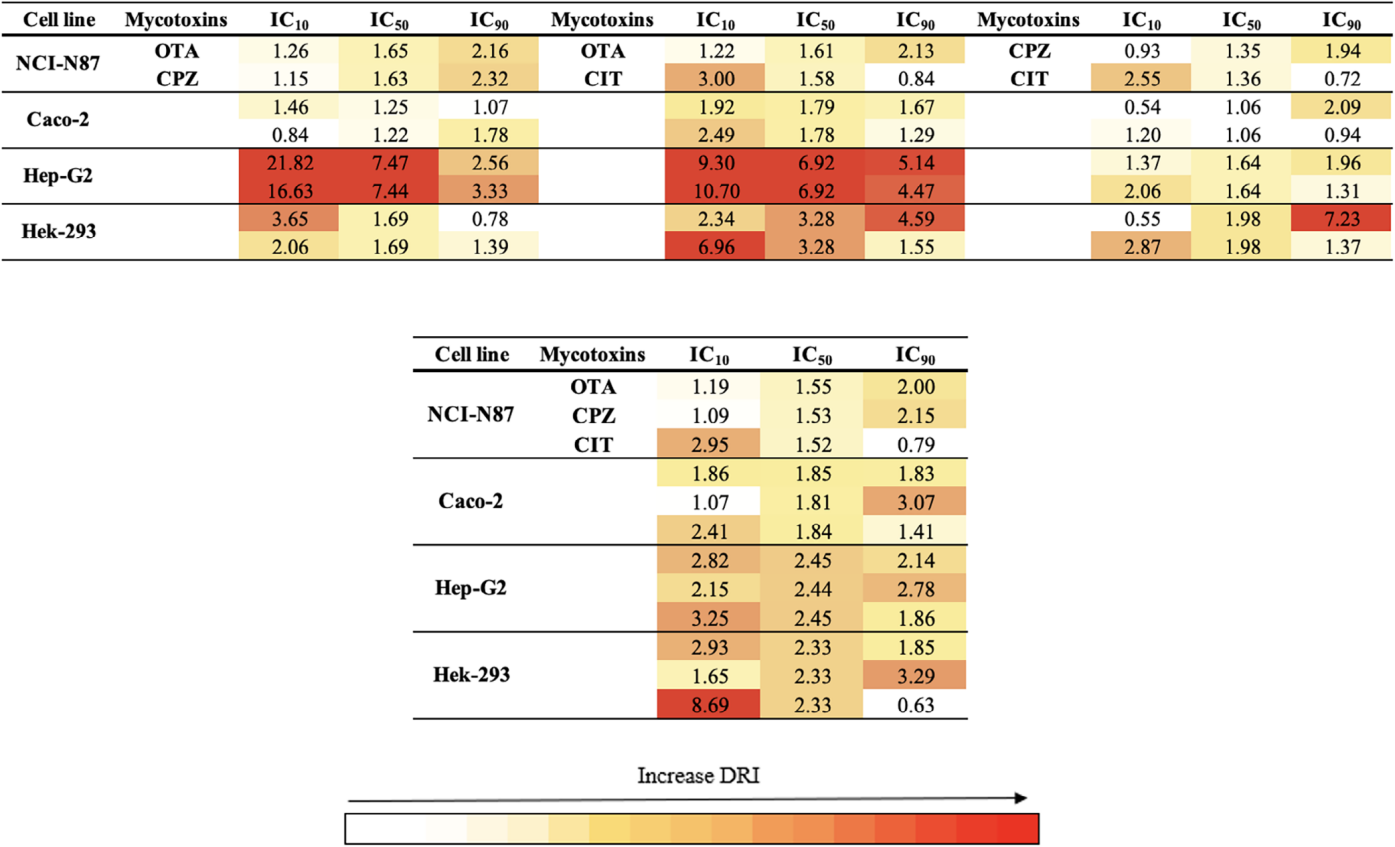
Dose reduction index (DRI) results for binary/ternary combinations at inhibition concentrations of 10%, 50%, and 90%. DRI < 1 indicates an unfavorable dose reduction; DRI = 1 indicates no dose reduction; DRI > 1 indicates a favorable dose reduction. CIT, citrinin; CPZ, cyclopiazonic acid; OTA, ochratoxin A; IC_10_, inhibition concentration that inhibits 10% of the cell population; IC_50_, inhibition concentration that inhibits 50% of the cell population; IC_90_, inhibition concentration that inhibits 90% of the cell population.

Apart from the degree of individual toxicity and type and magnitude of toxicological interactions, other relevant data computed in this study included the DRI, which discloses the extent to which the dose of each compound in the combination can be decreased to attain an equivalent level of inhibition compared to individual compound doses. Since the study was evaluating toxic effects, a DRI < 1 indicates a favorable dose reduction; DRI = 1 indicates no dose reduction; DRI > 1 indicates a non‐favorable dose reduction (Fig. 5). For the OTA:CPZ combination, synergistic effects were observed at all inhibition levels only in liver cells, with DRI values ranging from 2.56 to 21.82 for OTA and 3.33 to 16.63 for CPZ. Combined toxicity was also relevant in kidney cells, showing additive effects at lower inhibition levels and resulting in DRI values between 0.78 and 3.65 for OTA and 1.39–2.06 for CPZ. This combination was the most toxic in all cell lines (lower IC_50_) and was where the highest DRI value was found (21.82). The OTA:CIT combination had high synergic effects among the different cell types, which were more prominent in the liver and kidneys, so the highest DRI values were observed in these cells. Among all inhibition levels, the DRI values in the liver cells were between 5.14 and 9.30 for OTA and 4.47 and 10.70 for CIT, while in the kidney cells they were 2.34–4.59 for OTA and 1.55–6.9 for CIT. Regarding the CPZ:CIT combination, the only relevant DRI value found was in the high inhibition levels (IC_90_), with 7.23 fold for CPZ. To sum up, higher DRI values were observed in HepG2 and HEK‐293 when OTA, a regulated and highly prevalent mycotoxin, was compared with CPZ or CIT, the emergent ones. In general, the highest DRI values were observed at low inhibition levels (IC_10_), suggesting that the combinations may have distinct synergistic strength, which is particularly significant.

Most studies reported the potential toxic effect on individual mycotoxins, and few reported their combined effects. The results of the current study with regard to OTA:CIT align with previous reports of synergism in HepG2 and HEK‐293 cells. However, in this study, the IC₅₀ values of 12.05 μM (1.17 μM for OTA and 10.88 μM for CIT) in HepG2 and 13.27 μM (0.46 μM for OTA and 12.80 μM for CIT) in HEK‐293 indicate higher toxicity than those previously reported in HepG2, 42 μM for OTA and 31 μM for CIT, and in HEK‐293, 7 μM (equimolar concentration).^41^, ^44^ Differences in exposure time, cell density, and toxin ratios may explain these discrepancies. No prior data exist on the toxicity of the other binary combinations, as far as the authors are aware, making this study the first to report such results.

The results are relevant in the context of human risk, as dietary mycotoxin exposure typically occurs chronically and at low levels. In this study, predictive concentrations required to induce 10% inhibition (IC_10_) were calculated for individual and combined exposures, incorporating DRI values for binary and ternary combinations derived from the Chou–Talalay analysis. These values were then compared with the concentration range reported in foods (Table 3).^49^, ^50^, ^51^, ^52^, ^53^, ^54^, ^55^, ^56^ Although expressed in different units (mg L⁻¹ for *in vitro* exposure and mg kg⁻¹ for food occurrence), this comparison provides an indicative assessment of exposure relevance.

**Table 3:** Predictive concentration of the mycotoxins, individually and in binary and ternary combinations at inhibition concentrations (IC) of 10% and occurence range in food described in the literature^49^, ^50^, ^51^, ^52^, ^53^, ^54^, ^55^, ^56^

	Occurence range in food	Individual	Binary (OTA:CPZ)	Binary (OTA:CIT)	Binary (CPZ:CIT)	Ternary	Cell line
OTA	0.001–0.614	1.13	0.90	0.93		0.94	NCI
CPZ	0.025–3.61	1.46	1.27		1.57	1.34	
CIT	0.00023–44.24	11.89		3.96	4.66	4.03	
OTA		0.52	0.36	0.27		0.28	Caco‐2
CPZ		0.50	0.60		0.93	0.47	
		14.13		5.68	11.75	5.86	
OTA		1.12	0.05	0.12		0.40	HepG2
CPZ		0.79	0.05		0.58	0.37	
CIT		8.24		0.77	3.99	2.54	
O		0.12	0.03	0.05		0.04	HEK‐293
CPZ		0.45	0.22		0.82	0.27	
CIT		6.29		0.90	2.19	0.72	

All values are in mg kg^−1^ (foods) or mg L^−1^ (cell exposure medium). For binary and ternary combinations, the dose index reduction (DRI) was taken into account.

Abbreviations: CIT, citrinin; CPZ, cyclopiazonic acid; OTA, ochratoxin A.

Notably, several predicted IC_10_ values were of the same order of magnitude as concentrations reported in food matrices, even without accounting for synergistic interactions. When synergism was considered, the effective concentrations required to induce cytotoxicity decreased substantially. In HepG2 cells, the OTA:CPZ combination showed strong synergism (DRI of 21.82 and 16.63 for OTA and CPZ, respectively), reducing the predicted IC₁₀ to 0.05 mg L⁻¹ for each compound. In HEK‐293 cells, the IC₁₀ values for this combination decreased to 0.03 mg L⁻¹ for OTA and 0.22 mg L⁻¹ for CPZ.

A comparable effect was observed for the OTA:CIT combination. In HEK‐293 cells, synergistic interactions (DRI of 2.34 and 6.69 for OTA and CIT, respectively) reduced the predicted IC₁₀ values to 0.05 mg L⁻¹ for OTA and 0.90 mg L⁻¹ for CIT. In HepG2 cells, stronger synergism (DRI of 9.30 for OTA and 10.70 for CIT) resulted in predicted IC₁₀ values of 0.12 mg L⁻¹ for OTA and 0.77 mg L⁻¹ for CIT.

These results illustrate how synergistic interactions may lower effective toxicity thresholds substantially, and they highlight the potential relevance of mixture exposure under realistic contamination scenarios.

European regulations and recommendations have established maximum levels for individual mycotoxins in food and feed, focusing solely on single‐compound exposure scenarios.^57^ However, the co‐occurrence of mycotoxins in food commodities is widely documented, reflecting their widespread presence across agricultural products.^49^, ^50^, ^51^, ^52^, ^53^, ^54^, ^55^, ^56^, ^58^, ^59^, ^60^ In this context, mixture toxicity becomes particularly relevant, as interactions between co‐occurring mycotoxins may alter toxicological thresholds compared with individual exposures. The results presented in Table 3 illustrate this concern, showing that synergistic interactions can lower predicted IC_10_ values substantially when DRI are considered. As IC_10_ represents a low effect level commonly associated with early biological responses under chronic exposures conditions, these reductions suggest that mixture effects may occur at concentrations approaching those detected in contaminated foods.

Biomonitoring studies further indicate the importance of these exposures. Ochratoxin A has been detected frequently in human biological matrices, including blood, plasma, and urine, reflecting widespread dietary exposure.^61^ Although these levels are lower than the predicted IC₁₀ values obtained in the present *in vitro* study, the long biological half‐life of OTA (several weeks) and its strong binding to serum albumin contribute to sustained systemic exposure and potential tissue accumulation.^47^, ^48^, ^61^ Similarly, exposure to CIT has been confirmed through detection of its biomarker, dihydrocitrinone, in human urine, indicating ongoing intake in the general population.^62^, ^63^, ^64^ These results indicate that mycotoxins such as OTA and CIT are not only present in food but also reach measurable concentrations in the human body, reflecting real internal exposure. Consequently, the observation that synergistic interactions may lower cytotoxic thresholds toward concentrations reported in food, supported by biomonitoring evidence, highlights the potential toxicological relevance of combined exposure.

This observation aligns with EFSA guidance recognizing the importance of assessing combined exposure to multiple chemicals rather than assuming independent effects.^24^ The potential for synergistic interactions to reduce effective toxicity thresholds may therefore challenge traditional TDI frameworks, which rely on dose additivity assumptions. Within the broader framework of the human exposome, these findings highlight how chronic exposure to complex mixtures of food contaminants may contribute to cumulative biological effects that are not fully captured by single‐compound risk assessments.

### Cytotoxicity of ternary combinations of mycotoxins

All ternary combinations produced dose‐dependent reductions in cell viability across all cell lines, with stronger effects at higher concentrations (Fig. 2). Table 2 presents the parameters (*r, m*, and *D_m_*) of the dose–response curves, together with the concentrations required to reduce cell viability by 10% (IC₁₀), 50% (IC₅₀), and 90% (IC₉₀).

Gastric, intestine, and hepatic cells only exhibited antagonist effects, whereas renal cells showed a near‐additive response at low levels of inhibition (Fa < 0.2) (Fig. 3). Based on IC₅₀ values, toxicity followed the order HEK‐293 > HepG2 > NCI‐N87 > Caco‐2. The organ‐specific toxicity profile shifted for the ternary mixture. Ochratoxin A and CIT individually affected renal cells primarily, and CPZ affected intestinal cells primarily; however, the ternary combination predominantly affected renal cells. In contrast to binary combinations, where OTA:CIT and OTA:CPZ showed higher toxicity in hepatic cells, the ternary mixture altered the toxicity profile and reduced overall potency. The synergistic effects observed for OTA:CIT and OTA:CPZ in hepatic and renal cells were notably absent in the ternary combination.

The DRI values for ternary combinations were similar to those for binary combinations in gastrointestinal cells. In HEK‐293 cells, however, a significant DRI value was observed for CIT (8.69) (Fig. 5). The highest DRI values were observed in renal cells, followed by hepatic, intestinal, and gastric cells, and were most pronounced at low inhibition levels.

Citrinin is widely reported in the literature as one of the most frequently detected mycotoxins in cereal‐based products, often occurring at concentrations of up to hundreds of mg kg⁻¹.^58^ Consequently, the values reported in this study for combinations involving CIT are plausible (Table 3), particularly in light of the observed interaction effects.

This study provided novel qualitative and quantitative insights into the individual and combined cytotoxicity of OTA, CIT, and CPZ, supported by a controlled and reproducible experimental framework using immortalized human cell lines. This approach enables precise evaluation of cytotoxic effects and interactions under defined conditions. However, several limitations should be acknowledged. Although suitable for comparative analyses, these models do not fully capture the complexity of whole tissues, including three‐dimensional architecture, immune responses, microbiota interactions, and systemic regulation. Consequently, the findings primarily reflect cellular‐level responses rather than fully integrated organism‐level effects.

The concentrations tested were in the micromolar range and, at the highest levels, may exceed reported circulating plasma concentrations following dietary exposure. However, the data remain relevant when low‐effect thresholds (e.g., IC₁₀), as discussed above, are considered. In an intestinal tract model system, higher transient local concentrations may also occur prior to absorption and systemic metabolism. Although the constant‐ratio mixture design applied here is appropriate for initial interaction analyses, it does not fully reflect the variability of mycotoxin ratios in real food matrices, highlighting the need for further investigation.

## CONCLUSION

The consequences of mycotoxin contamination extend beyond environmental concerns to impact human health directly. Mycotoxins contamination represents an important food safety concern, particularly due to the frequent co‐occurrence of multiple compounds in food commodities. This study assessed the individual and combined toxicity of three *Penicillium* mycotoxins – OTA, CIT, and CPZ – using human cell models representing key physiological barriers and organs involved in xenobiotic absorption (NCI‐N87, Caco‐2), metabolism (HepG2), and excretion (HEK‐293). Ochratoxin A was the most toxic across all cell lines, with pronounced effects in renal and intestinal cells. Cyclopiazonic acid mainly impacted intestinal cells, and citrinin showed moderate toxicity, predominantly in renal cells.

Combination analysis revealed that binary mixtures can modify toxicological responses substantially. Synergistic interactions were observed, particularly for OTA:CPZ in hepatic cells and OTA:CIT in hepatic and renal models, even at low effect levels (IC_10_). These interactions were associated with high DRI values, indicating that lower concentrations of each compound are required to induce cytotoxic effects when combined. In contrast, the ternary mixture showed additive effects in renal cells and antagonistic effects in other cell types, suggesting that mixture complexity may alter interaction patterns and organ‐specific toxicity.

These findings emphasize the need to assess co‐exposure to multiple mycotoxins in food safety risk assessment. A clear research gap remains regarding low‐level exposure to mycotoxins and the co‐occurrence of multiple mycotoxins. Current regulatory frameworks focus on individual compounds, whereas real‐world exposure involves complex mixtures. Future research should prioritize elucidation of the molecular mechanisms underlying these interactions, improved integration of mixture toxicity data into cumulative risk assessment frameworks, and alignment of *in vitro* findings with biomonitoring and exposure data to better characterize the health implications of multi‐mycotoxin exposure.

## FUNDING INFORMATION

This work was supported by national funds from Portugal through the Fundação para a Ciência e Tecnologia and Ministério da Educação, Ciência e Inovação (FCT/MECI, Foundation for Science and Technology and Ministry of Education, Science and Innovation). Funding was provided under the following projects: UID/50006/2025 (https://doi.org/10.54499/UID/50006/2025) – Laboratório Associado para a Química Verde – Tecnologias e Processos Limpos (Associated Laboratory for Green Chemistry – Clean Technologies and Processes); UID/04423/2025 (https://doi.org/10.54499/UID/04423/2025); UID/PRR/04423/2025 (https://doi.org/10.54499/UID/PRR/04423/2025); and LA/P/0101/2020 (https://doi.org/10.54499/LA/P/0101/2020).

## CONFLICT OF INTEREST

The authors declare no conflict of interest.

## Data Availability

The data that support the findings of this study are available from the corresponding author upon reasonable request.
